# An Overview of Direct Somatic Reprogramming: The Ins and Outs of iPSCs

**DOI:** 10.3390/ijms17010141

**Published:** 2016-01-21

**Authors:** Siddharth Menon, Siny Shailendra, Andrea Renda, Michael Longaker, Natalina Quarto

**Affiliations:** 1 Hagey Laboratory for Pediatric Regenerative Medicine, Department of Surgery, School of Medicine, Stanford University, 257 Campus Drive, Stanford, CA 94305, USA; smenon8@stanford.edu (S.M.); sinys@stanford.edu (S.S.); 2 Dipartimento di Scienze Biomediche Avanzate, Universita’ degli Studi di Napoli Federico II, Napoli 80131, Italy; renda@unina.it; 3 Institute for Stem Cell Biology and Regenerative Medicine, School of Medicine, Stanford University, Stanford, CA 94305, USA

**Keywords:** embryonic stem cells, adult stem, somatic reprogramming, induced pluripotent stem cells (iPSCs)

## Abstract

Stem cells are classified into embryonic stem cells and adult stem cells. An evolving alternative to conventional stem cell therapies is induced pluripotent stem cells (iPSCs), which have a multi-lineage potential comparable to conventionally acquired embryonic stem cells with the additional benefits of being less immunoreactive and avoiding many of the ethical concerns raised with the use of embryonic material. The ability to generate iPSCs from somatic cells provides tremendous promise for regenerative medicine. The breakthrough of iPSCs has raised the possibility that patient-specific iPSCs can provide autologous cells for cell therapy without the concern for immune rejection. iPSCs are also relevant tools for modeling human diseases and drugs screening. However, there are still several hurdles to overcome before iPSCs can be used for translational purposes. Here, we review the recent advances in somatic reprogramming and the challenges that must be overcome to move this strategy closer to clinical application.

## 1. Introduction

Stem cells are a subset of cells in our body with the remarkable ability to self-renew and differentiate along different cell-lineages [1]. They can be classified into two main categories based on their self-renewing capacity and plasticity, namely “embryonic stem cells” and “non-embryonic” adult/somatic stem cells. Self-renewal refers to the ability to undergo multiple divisions while maintaining an undifferentiated state, while plasticity refers to the ability of a cell to differentiate down multiple different cell lineages. Plasticity can also be referred to as the potency of a cell.

**Figure 1:**
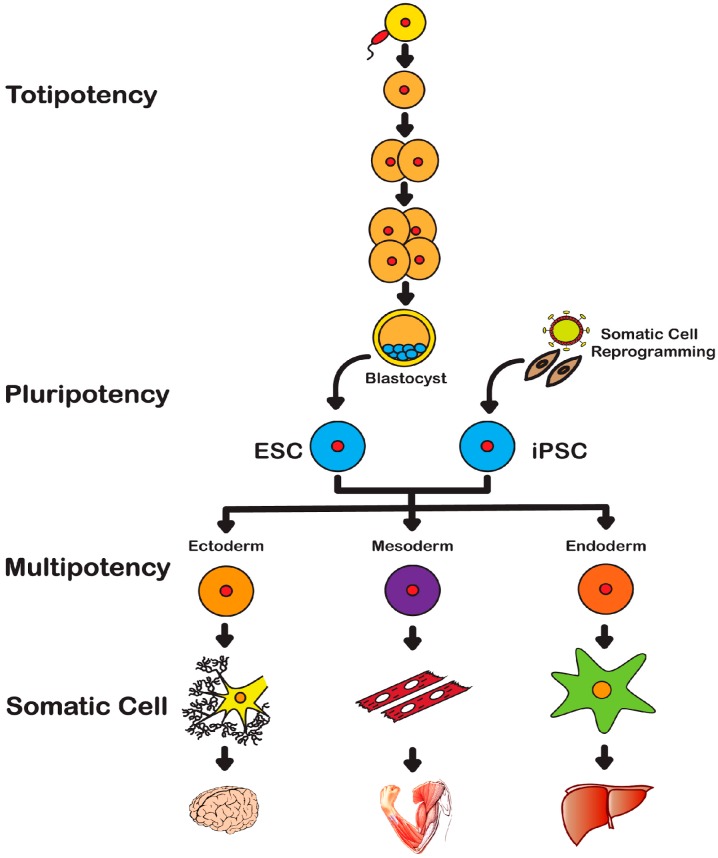
Totipotency: After fertilization, Embryonic Stem Cells (ESCs) maintain the ability to form all three germ layers as well as extra-embryonic tissues or placental cells and are termed as totipotent. Pluripotency: These more specialized cells of the blastocyst stage maintain the ability to self-renew and differentiate into the three germ layers and down many lineages but do not form extra-embryonic tissues or placental cells. Reprogrammed somatic cells, iPSCs, also demonstrate the ability to self-renew and differentiate into all three germ layers *in vivo* and *in vitro*. Thus, iPSCs are also considered to be pluripotent stem cells. Multipotency: Adult or somatic stem cells are undifferentiated cells found in postnatal tissues. These specialized cells are considered to be multipotent; with very limited ability to self-renew and are committed to lineage specific differentiation.

Embryonic Stem Cells (ESCs) have the unique potential to endlessly divide while maintaining an undifferentiated state (self-renewing) but also the capacity to differentiate into all germ layers as well as extra-embryonic tissues or placental cells, being termed as totipotent. Days after fertilization, these totipotent cells mature and form more specialized cells called pluripotent cells (Figure 1). Pluripotent stem cells maintain the ability to self-renew and differentiate into all three germ layers and down many lineages. These cells, through complex mechanisms are responsible for tissue growth, repair and maintenance. The so-called mouse ESCs (mESCs) are isolated at day E3.5 from the inner cell mass of blastocyst [2] whereas human ESCs (hESCs) are isolated from late blastocyst and correspond to the epiblast stem cells of the mouse [3]. Cell lines derived from mouse epiblast share defining features with hESCs [4]. These *in vitro* derived stem cells are pluripotent and due to their highly regenerative capacity, hESCs are a strong candidate for cell-based therapies, drug studies and disease modeling. However, advances in embryonic stem cell technologies are limited by the controversial source and methods of isolation.

Non-embryonic adult/somatic cells are undifferentiated cells found in postnatal tissues. They are more specialized cells and are multipotent, in that they have very limited ability to self-renew, vary in their degree of plasticity (depending on the tissue origin) and are often committed to lineage specific differentiation. These more “mature” cells are referred to as adult or somatic stem cells due to their restricted ability to differentiate. Adult stem cells can be isolated from a variety of sources and are often named after their tissue of origin as follows: Bone Marrow Stem Cells (BMSCs), Adipose-derived Stem Cells (ASCs), Hematopoietic Stem Cells (HSCs) [5,6,7].

The more recent discovery of induced Pluripotent Stem Cells (iPSCs) may circumvent the several ethical issues and moral conflicts associated with the use of hESCs in clinical applications [8]. Several reports have indicated that iPSCs share features with hESCs both *in vitro* and *in vivo*, such as the ability to self-renew and differentiate into many cell types. Indeed, the potential use of iPSCs in regenerative medicine is reassuring for the ethical concerns associated with hESCs and provide potentially an unlimited source of cells for many therapeutic and discovery based applications.

In this review, after a brief introduction on the history and discovery of ESCs, we will mainly focus on the current derivation procedures, applications and future perspectives of iPSCs; their limitations will be discussed as well.

## 2. History of Stem Cells

### 2.1. Embryonic Stem Cells

Mouse ESCs were the first pluripotent cell type isolated from early embryos. In 1981, Evans and Kaufman [1] and Gail R. Martin [2], independently, described the successful establishment of mESC cultures derived from mouse blastocysts. Since these initial reports of mESCs, derivation of ESC lines has been attempted in several non-rodent species with little success [9]. In 1998, Thomson and colleagues derived hESCs from blastocyst of human embryos produced by *in vitro* fertilization (IVF) for clinical purpose [3] thus, establishing the first cultured human embryonic stem cells line. Thomson proposed three criteria that defined the primate ESCs: (i) derivation from the pre-implantation or peri-implantation embryo; (ii) prolonged undifferentiated proliferation and iii. STABLE developmental potential to form derivatives of all three embryonic germ layers even after prolonged culture [3]. These valuable properties make hESCs an ideal tool for regenerative medicine, cell therapy and drug discovery. However, their controversial derivation from the cleavage stage of human embryonic tissue has proven to be a significant obstacle in the advancement of embryonic stem cell technologies.

### 2.2. Induced Pluripotent Stem Cells

Pluripotency can also be re-instated in cells of later developmental stages through specific techniques. In 1958, Gurdon *et al.* [10] using the technique of nuclear transplantation, originally described by Briggs and King [11], showed that the nuclei of intestinal epithelial cells from feeding tadpoles, after transplantation into enucleated eggs, could develop into normal and healthy tadpoles, thus demonstrating successful nuclear reprogramming. This first somatic cell nuclear transfer (SCNT) suggested the presence in ESCs of key-factors inducing and/or maintaining pluripotency. This discovery laid the groundwork for future breakthroughs and achievements in cellular reprogramming.

In 2006, Yamanaka and Takahashi [8] demonstrated the ability to induce a pluripotent state in somatic cells through retroviral-mediated ectopic expression of four genes: *Octamer-Binding Transcription* *Factor 4* (*Oct4*), SRY (*Sex Determining Region Y*)*-box 2* (*Sox2*), *Kruppel-Like Factor 4* (*Klf4*) and *Avian* *Myelocytomatosis Viral Oncogene Homolog* (*c-Myc*). These four genes are known also as the “Yamanaka Factors” or OSKM factors. The generation of iPSCs with OSKM has been described as “direct” reprogramming in contrast to reprogramming via nuclear transfer. In addition to Nobel Prize recognition, the discovery of iPSCs has also encouraged scientists to look beyond hESCs for regenerative medicine purposes. These artificially derived pluripotent cells exhibit molecular and functional characteristic similar to those of the embryonic epiblast, and unlike hESCs, iPSCs are not controversial in their source. Moreover, they can potentially provide unlimited autologous cells for cell-based therapies, modeling human diseases, identifying new therapeutic targets and testing new therapies. We will discuss below current reprogramming methods and strategies.

## 3. Reprogramming Techniques

**Figure 2:**
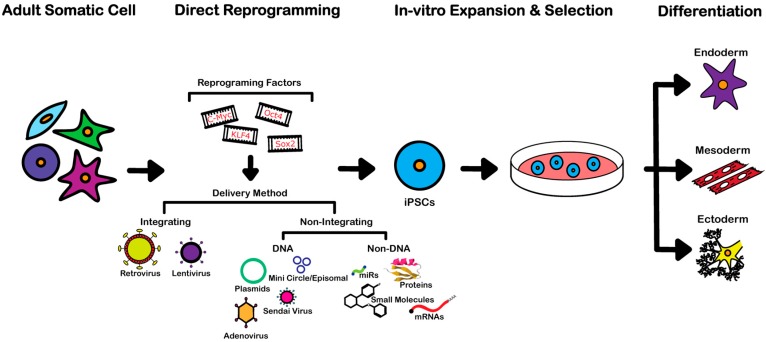
The direct reprogramming of somatic cells to induced pluripotent stem cells (iPSCs) can be achieved through the ectopic expression of specific transcription factors: *Oct-4*, *C-Myc*, *Sox-2* and *Klf-4*, the “Yamanaka or OSKM Factors”. Delivery of these factors can be accomplished through a variety of methods. Initial methods developed integrating retroviral or lentiviral vectors. More recent strategies utilized non-integrating methods, further categorized by the use of DNA such as plasmids, mini circles/episomal, adenovirus, Sendai virus, and non-DNA based procedures such as, mRNAs, microRNAs (miRs), small molecules and bioactive proteins. Once reprogrammed to a pluripotent state, iPSCs can be expanded *in vitro* and subsequently differentiated to ectoderm, mesoderm and endoderm linages for use in cell therapy, disease modeling and drug discovery.

A variety of somatic cell types included fibroblasts, blood, keratinocytes, liver and gastrointestinal cells, as well cancer cells, can be used to derive iPSCs [8,12,13,14,15,16,17,18,19,20,21,22]. As previously mentioned, the initial proof-of-concept studies on generation of mESC-like cells were performed using retroviral transduction of mouse fibroblasts with four specific transcription factors [8]. Currently, several methods exist for generating iPSC lines, but those best suited for studying human diseases and developing therapies must be of satisfactory efficiency and fulfill needs such as generation of iPSCs from samples that may be of limited abundance, ability of reprogramming from different somatic cells, and footprint-free. To date, several reprogramming techniques meet these criteria and are readily employed to derive iPSCs (Figure 2).

### 3.1. Integrating Procedures

The first generation of iPSCs was achieved by retroviral transduction technique of OSKM factors into mouse fibroblasts [8]. This method of direct reprogramming of somatic cells to iPSCs utilizes a retroviral-mediated ectopic expression of OSKM identified by Yamanaka and Takahashi through rigorous screening of 24 factors associated with pluripotency. Retroviral transduction to derive iPSCs has been successfully used for several cell types, such as mouse and human fibroblasts, neural stem cells, keratinocytes, adipose cells, liver cells and blood cells. The reprogramming efficiency obtained using human cells is between 0.01%–0.02% [13]. An alternative approach to transduce OSKM factors to derive iPSCs is the use of a lentiviral system which yields a higher efficiency (0.1%–2%) than retroviral transduction [23]. Although the discovery of Yamanaka factors using retroviral and/or lentiviral systems provides an alternate way to obtain embryonic-like stem cells in limitless quantity, it does have significant drawbacks. The disadvantage of viral integration into the host genome as well as the pro-cancerous role of c-Myc in malignant transformation limit the translational application of iPSCs lines derived in this manner [23,24,25]. Subsequently, from a clinical perspective, direct reprogramming through retroviral and lentiviral transduction of OSKM factors is not yet translational and various other reprogramming methods have gained popularity.

### 3.2. Non-Integrating DNA-Based Procedures

As previously stated, a major shortcoming of the initial reprogramming strategies is the integration of viral vectors used to transduce the reprogramming factors into host chromosomes. Integration can cause insertional mutagenesis, interference with gene transcription, genome instability and induce malignant transformation [26]. For instance, studies in mice demonstrated iPSC-derived chimeras frequently develop tumors resulting from reactivation of the oncogene c-Myc [27,28]. Although direct reprogramming has been achieved without c-Myc, it has been shown that the three remaining integrated reprogramming factors could also induce tumors [26,29]. Studies report that retroviral infection leads to an average 10–20 retroviral integration sites in human iPSCs lines [13,30]. Therefore, in order to employ iPSCs for human therapy, their reprogramming should be done using non-integrating strategies. In light of translational goals, several non-integrating virus-mediated iPSCs reprogramming methods have been currently developed.

The development of new techniques such as the transgene excision method, circumvent the potential risks caused by the integration of viral vectors. Recently, several studies have reported alternatives to retroviral integration methods [23,25,31]. One example is a doxycycline (DOX)-inducible lentiviral vector, harboring OSKM factors that can be excised with Cre-recombinase allowing for a differential expression of these reprograming factors, in addition to transfecting both dividing and non-dividing cells, increasing reprogramming efficiency. The first attempt to derive iPSCs without transgene integration used lentiviral vectors containing LoxP sites introduced into the 5′ and 3′ LTR. These LoxP sites provide means of nearly excising all transgene through Cre-mediated recombination [32]. Cre-mediated recombination can be achieved either using picornaviral 2A plasmids or adenoviral Cre. Upon Cre recombination, most of the transgene is excised but a potential risk of insertional mutations from residual vector sequences may not be ruled out. Thus, from a clinical perspective, even minimal residual vector sequences would be a cause for concern when these iPSCs are differentiated and subsequently transplanted into a patient.

The delivery of polycistronic reprogramming OSKM factors expression cassettes with PiggyBack (PB) transposon into somatic cells is another non-integrating method of the simplest and most robust reprogramming approaches representing a low risk reprogramming procedure [33]. This technique utilizes a mobile genetic element that efficiently transposes between vectors and chromosomes with no integration. The PB transposase recognizes specific inverted terminal repeat sequences (ITR) located on either ends of transposon and efficiently integrates them to TTAA sequence chromosomal sites. Woltjen *et al.* [34] demonstrated successful transposon-based reprogramming of fibroblasts to iPSCs in murine cells with clean excision of vector sequence. Kaji *et al.* [35], combining a non-viral transfection of a single polycistronic expression vector, harboring the coding sequences of OSKM, with a piggyback transposon procedure succeeded in establishing reprogrammed human cell lines from embryonic fibroblasts. The derived iPSCs expressed robust levels of pluripotency markers and were generated with an efficiency of 0.02%–0.05%. Although, there have been reports of successful human iPSCs derived using PB transposons, it must be further demonstrated that the transposons are fully removed prior to employing this procedure for translational purposes. However, some studies suggest that the removal of a large number of transposon copies is difficult to achieve [23,36].

Replication-defective adenoviral vectors expressing OSKM factors have proven useful for derivation of iPSCs because they do not integrate into chromosomal DNA [37,38]. Although they are non-integrating, gene expression can last for days, thus providing sufficient time to reprogram somatic cells to pluripotency. Adenoviral vectors have been largely used to generate iPSCs from liver cells and fibroblasts without viral integration [31,39]. These vectors have lower transduction efficiency in comparison to their retroviral counterparts [31]. While the non-integrating aspect of the adenoviral method is appealing, to be of significant use in translational medicine, optimization of efficiency is necessary.

Non-viral minicircle DNA vectors containing Lin28, GFP (Green Fluorescent Protein), Nanog, Sox2, and Oct4 transcripts have been described as a procedure to derive human iPSCs from human adipose stromal cells with an efficiency of 0.005% [40]. This method, although non-integrating, lacks sufficient validation to implement in translational studies.

Episomal plasmids provide another method for integration-free reprogramming of somatic cells into iPSCs [41,42]. This procedure has also been used to derive iPSCs from cord blood and peripheral blood cells [43]. This technique yields a very low efficiency, but several modifications by different groups provide promising results for future use [41,42,44].

### 3.3. Non-Integrating Non-DNA-Based Procedures

The single-stranded RNA Sendai virus (SV) is also an attractive option for iPSCs derivation, as genomic material does not enter the nucleus of the host cell, does not integrate into the host genome and can be easily removed by antibody-mediated negative selection [45]. Work by Fusaki *et al.* [45] reported the effective induction of human iPSCs from fibroblasts using Sendai virus as a safe alternative to integrating retroviruses. Sendai virus is potentially a strong candidate for translational research as the efficiency of iPCSs derivation obtained using this procedure is approximately 0.1%, comparable to the lentivirus while remaining non-integrating. Although they are difficult to work with as compared to the lentivirus, commercially available Sendai viral extracts make this a promising alternative procedure.

Another way to avoid the introduction of genetic integrating material into donor cells for their reprogramming to iPSCs is mRNA transfection [46]. RNA-induced pluripotent stem cells (RiPSCs) procedures offer a safe and effective method to generate iPSCs with “null footprint”. Additionally, this method of somatic cell reprogramming using synthetic mRNA provides a reduced immunogenic response. Warren *et al.* [47] demonstrated that repeated administration of synthetic modified messenger RNAs encoding for the OSKM factors, and designed to bypass innate antiviral responses, can reprogram several differentiated human cells to pluripotency with conversion efficiencies and kinetics substantially superior to established viral protocols. Using these methods, Warren *et al.* [47] derived iPSCs from human keratinocytes, BJ human neonatal fibroblasts, MRC-5 human fetal lung fibroblasts, and cystic fibrosis patient fibroblasts. Although this procedure can reprogram cells at an efficiency of more than 2%, it suffers from major drawbacks: it is time consuming and labor intensive.

The sole expression of specific microRNAs (miRs) clusters also represents a suitable non-integrating, non-DNA-based method to reprogram both mouse and human somatic cells into iPSCs. Miyoshi *et al.* [48], derived iPSCs by transfecting with a combination of mature miRs clusters, specifically, mir-200c, mir-302s and mir-369s family miRs. These reprogramming miRs were identified upon a large analysis of miRs expression in mouse ESCs, mouse iPSCs, and adult mouse adipose stromal cells. Using this approach several mouse and human iPSCs lines have been derived [49]. Because this reprogramming method does not require vector-based gene transfer, it holds significant potential for regenerative medicine.

Purified OSKM proteins represent another appealing “non-DNA” reprogramming method. Bioactive OSKM proteins at first glance seem as if they are the perfect choice for reprogramming without integrating into host genome [50]. Kim *et al.* [51] demonstrated generation of stable iPSCs from human fibroblasts by direct delivery of four reprogramming protein factors (OSKM) yielding an efficiency of 0.001%. The human iPSC lines produced with these recombinant proteins (p-iPSCs) were successfully maintained for more than 35 passages and differentiated into derivatives of all three embryonic germ layers both *in vitro* and *in vivo* [51]. A major challenge of this procedure is the intracellular delivery of OSKM proteins due to their limited ability to cross the cellular membrane [52]. This can be overcome by fusing them to a short basic segment, namely Cell Penetrating Peptide (CPP), containing a high proportion of basic amino acids (e.g., arginine and lysine) [53,54]. Several studies have reported that CPP-anchored reprogramming OSKM proteins, when delivered into somatic cells, can directly reprogram them successfully without genetic manipulation and/or chemical treatments [50,51]. However, despite successful induction of pluripotency, bioactive reprogramming proteins are difficult to synthesize in large quantities, and efficiency varies between 0.001%–4%.

Cellular reprogramming using small molecules may also represent an attractive and convenient alternative to transcription factor-mediated lineage reprogramming. This procedure offers many advantages such as temporally and spatially controllable, reversible, tunable, cell permeability and cost effectiveness. Small molecules used to generate iPSCs are comprised of epigenetic modifiers, WNT signal modulators, cell senescence attenuators, metabolism modulators, and regulators of cell apoptosis/senescence pathways. Small molecules inducing iPSCs can be classified into three types: (1) small molecules that improve reprogramming efficiency [55]; (2) compounds replacing one or more reprogramming factors [56,57,58]; and (3) compound cocktails alone that are sufficient to induce iPSCs [59,60]. Small molecule methods have been successfully applied to reprogram mouse fibroblasts directly into functional neurons using only a combination of small molecules [59,60]. Induced neural progenitor cells (iNPCs) have also been generated using a chemical cocktail comprised of an inhibitor of GSK-3 kinases and TGF-β pathways under physiologically hypoxic conditions [61]. Although several studies have demonstrated that small-molecule-direct reprogramming can completely replace the ectopic OSKM factors in reprogramming into neural lineages, many other studies have combined ectopic transcriptional factors with small molecules to derive neuronal lineages from somatic cells [61,62,63]. Indeed, small molecule-induced iPSC have dramatically changed iPSC research as highlighted by several studies showing that some of these small molecules increase reprogramming efficiency and quality, while others (or combinations of them) can fully replace OSKM reprogramming factors.

## 4. Clinical and Therapeutic Application of iPSCs

### 4.1. Tissue Regeneration and Cell Transplantation Therapy

Generation of autologous cells for tissue regeneration and cell transplantation therapy has been the elusive goal of regenerative medicine since the discovery and establishment of the first pluripotent stem cells. Their inherent regenerative properties make them the ideal candidate for cell-based therapies. The self-renewing capacity in combination with their plasticity, hESCs provide significant potential in translational medicine. Nevertheless, due to the controversial source, limitations in efficient differentiation protocols and immunological incompatibility between host and donor cells, the translational progress of hESCs has been limited in cell transplantation therapies. In recent years, the use of iPSCs as alternate sources of embryonic like stem cells has increased in popularity. The somatic cell origin, ease of *in vitro* expansion and absence of immunological response due to “personalized” cell lines (host derived donor cells) make iPSCs a very appealing alternative to the more controversial hESCs. It has been established that cardiomyocytes derived from human iPSCs are functionally equal to those derived from hESCs [64]. Additionally, they demonstrated the capacity of these iPSCs to differentiate into nodal-, atrial-, and ventricular like phenotypes providing a novel, autologous source of cells for cardiac repair and cardiovascular research. Zhang *et al.* [65] demonstrated the potential of embryonic stem cells in a rescue of a Hemophilia B mouse model. Due to limitations in transplantation efficiency (reported 0.03%–0.5%), need for recipient preconditioning and challenges in differentiation and expansion to functional hepatocytes from these mESCs, new methods using iPSCs were explored. iPSCs derived from wild-type mice were obtained and subsequently differentiated into hepatocytes producing coagulation factor IX (FIX) both *in vivo* and *in vitro*. The iPSCs derived hepatocytes functionally engrafted into the hepatic parenchyma showed improved FIX clotting activity by 2%–3%, demonstrating substantial improvement and potential disease phenotype rescue.

While the work of both Wu and Zhang provide compelling evidence for the application of iPSCs and the rescue of disease phenotypes, the work of Hanna and co-workers, in 2007 [66] demonstrated the true potential of iPSCs technology through a rescue of a humanized mouse model of sickle cell anemia using iPSCs derived hematopoietic stem cells. iPSCs were derived from tail-tip fibroblasts of mice affected by a hematopoietic genetic mutation modeling human sickle cell anemia. The subsequent iPSCs were then repaired through homologous recombination of the genetic defect. These corrected iPSCs were then expanded *in vitro* and differentiated to hematopoietic stem cells and finally transplanted into host mice. Correction of the defect was evaluated by electrophoresis for human beta globin proteins A and S (HBA and HBS). Significant levels of HBA were detected while levels of HBS had decreased at the four- and eight-week periods, indicating a rescue of the disease phenotype and restoration of normal hemoglobin levels.

### 4.2. Disease Modeling and Drug Screening

Definitively, iPSCs represent a potential unlimited source of autologous cells for regenerative medicine. It has been established that iPSCs derived from patients with known genetic disorders can produce cell lines phenocopying the genetic disorder profile. The use of iPSCs in disease modeling allows for the derivation of many cell lines from different organs and tissue sources from which disease etiology and potential drug therapy can be studied.

The large number of studies reporting the use of iPSCs for modeling disease demonstrates indeed, that these disease-specific stem cells offer a unique opportunity to recapitulate the pathologic human condition *in vitro*, thereby enabling disease investigation and drug development.

Disease-specific iPSC lines were first reported in 2008 from two groups. Dimos *et al.* [67] generated iPSCs from an 82-year-old woman diagnosed with a familial form of amyotrophic lateral sclerosis (ALS). A second study by Park *et al.* [68] described the successful derivation of iPSCs from patients affected by different genetic diseases with either a Mendelian or multifactorial inheritance. Since these first two reports, many other studies have demonstrated the valuable use of iPSCs either to modeling disease or screening drugs *in vitro*. Some examples are reported below and in Table 1.

**Table 1:** Examples of current, established iPSC lines derived from patient-specific somatic cells that recapitulate various disease phenotypes.

Disease Type	Disease	Cell Line Derived	Reference
Neurological	Huntington’s Disease (HD)	Fibroblasts	[69]
	Parkinson’s Disease (PD)	Fibroblasts	[70]
	Familial Alzheimer's Disease (AD)	Fibroblasts	[71]
	Frontotemporal Dementia (FTD)	Fibroblasts	[72]
	Amyotrophic Lateral Sclerosis (ALS)	Fibroblasts	[73,74,75]
	Spinocerebellar Ataxia Type 2 (SCA 2)	Fibroblasts	[76]
	Machado-Joseph Disease (MJD)	Fibroblasts	[77]
	Rett Syndrome	Fibroblasts	[78]
Cardiac/Muscular	LEOPARD Syndrome	Fibroblasts	[79]
	Duchenne Muscular Dystrophy (DMD)	Fibroblasts	[80]
	Long QT Syndrome	Fibroblasts	[81]
	Pompe Disease	Fibroblasts	[82]
	Arrhythmogenic Right Ventricular Cardiomyopathy (ARVC)	Fibroblasts	[83,84]
	Dilated Cardiomyopathy (CMD)	Fibroblasts	[85]
	Barth Syndrome (BTHS)	Fibroblasts	[86]
	Friedreich Ataxia	Fibroblasts	[87]
Metabolic	Alpha-1 Antitrypsin Deficiency (A1ATD)	Fibroblasts	[88]
	Familial Hypercholesterolemia (FH)	Fibroblasts	[89]
	Glycogen Storage Disease Type 1a (GSD1a)	Fibroblasts	[90]
	Glycogen Storage Disease Type 1b (GSD1b)	Fibroblasts, Hepatic Cells (nonparenchymal)	[91]
	Type 1 Diabetes	Fibroblasts	[92]
	Gauchers Disease (GD)	Fibroblasts	[93]
	Mitochondrial Encephalomyopathy, Lactic Acidosis, and Stroke-like Episodes (MELAS)	Fibroblasts	[94]
	Carnitine Palmitoyltransferase II (CPT II)	Fibroblasts	[95]
Eye/Retina	Usher Syndrome (USH)	Keratinocytes	[96]
	Retinitis Pigmentosa (RP)	Fibroblasts	[97,98,99]
	Leber Congenital Amaurosis (LCA)	Fibroblasts	[100]
	Gyrate Atrophy (GA)	Fibroblasts	[101]
	Best Vitelliform Macular Dystrophy (BVMD)	Fibroblasts	[102]
	Age Related Macular Degeneration (AMD)	T-Cells	[103]
Blood	Shwachman-Diamond Syndrome (SDS)	Fibroblasts	[104]
	Dyskeratosis Congenita (DKC)	Fibroblasts	[105]
	Familial Platelet Disorder (FPD)	Peripheral T Cells	[106]
	Sickle Cell Disease	MSCs	[107,108]
	β-Thalassemia	Fibroblasts	[109]
	Myeloproliferative Disorders (MPDs)	Peripheral Blood CD34^+^ Cells	[18]
	Myelodysplastic Syndromes (MDS)	Hematopoietic Cells	[17]
Skeletal/Bone	Fibrodysplasia Ossificans Progressiva	Fibroblasts	[110]
	Menkes Disease (MNK)	Fibroblasts	[111]
	Skeletal Dysplasia (SD)	Fibroblasts	[112]
	Marfan Syndrome (MFS)	Fibroblasts	[113]
	Craniometaphyseal Dysplasia (CDM)	Peripheral Blood Mononuclear Cells	[114]
Cancer	Pancreatic ductal Adenocarcinoma (PDAC)	Primary PDAC Cells	[115]
	Chronic Myeloid Leukemia (CML)	Primary CML Cells	[20,116]
	Juvenile Myelomonocytic Leukemia (JML)	Primary JML Cells	[117]
	Gastrointestinal Cancer	Gastrointestinal Cancer Cells	[21]
	Li-Fraumeni Syndrome (LFS)	Fibroblasts	[118]
Other	Hutchinson Gilford Progeria	Fibroblasts	[119]
	Primary Ovarian Insufficiency (POI)	Fibroblasts	[120]
	Hermansky-Pudlak (HP)	Fibroblasts	[121]
	Chediak-Higashi (CH)	Fibroblasts	[121]

iPSCs technology is providing exciting new opportunities in cardiovascular research by creating platforms to study the mechanisms of disease pathogenesis that could lead to new therapies. For instance, iPSCs derived from patients with LEOPARD syndrome, an autosomal-dominant developmental disorder caused by mutation in the PTPN11 gene encoding the SHP2 phosphatase, can recapitulate the hypertrophic cardiomyopathy phenotype of this disease [81]. Moretti *et al.* [83] derived patient-specific iPSCs from members of a family affected by long-QT syndrome type 1, an autosomal dominant mutation in *KCNQ1* gene. Long-QT syndrome is characterized by abnormally prolonged ventricular repolarization phase, ventricular tachycardia. iPSCs derived from long-QT patients could differentiate into functional cardiac myocytes and recapitulated the electrophysiological features of the disorder. These reprogramming approaches hold promise as a potential application for regenerative medicine as well as preclinical drug and toxicity screenings in the cardiac field.

Parkinson’s disease (PD) is a devastating and progressive neuro-degenerative disorder characterized by the loss of neurons in the peripheral and central nervous system. Mutation or duplication/triplications of the α-synuclein gene (SNCA) are associated with the familial autosomal forms of PD [122,123]. iPSC-derived midbrain dopaminergic neurons from PD patients, carrying the SNCA triplication, exhibit the signature disease-related phenotype, characterized by accumulation of α-synuclein and susceptibility to oxidative stress [70].

Furthermore, studies have shown iPSCs to recapitulate signature pathological phenotypes of inherited metabolic disorders of the liver [90]. Ghodsizadeh *et al.* [22] reported the first successful derivation of functional liver disease-specific iPSC lines from patients’ fibroblasts.

Successful examples of iPSCs modeling skeletal genetic diseases have been reported for Marfan syndrome (MFS), an autosomal dominant disorder of connective tissue caused by mutations in the gene coding for Fibrillin-1 (FBN-1). A study by Quarto *et al.* [113] demonstrated that iPSCs derived from MFS patient fibroblasts faithfully phenocopied the skeletogenic profile observed in hESC carrying the same monogenic FBN1 mutation. This study provided the first evidence that *in vitro* iPSC models can be used to dissect molecular mechanisms underlying MFS syndrome [124].

The dominantly inherited Skeletal Dysplasias (SDs) is characterized by abnormal chondrogenesis during cartilage growth plate differentiation caused by mutation of the *TRPV4* gene encoding the transient receptor potential vanilloid family member 4, calcium channel protein. iPSCs derived from SD patients can recapitulate dysregulated processes of aberrant chondrogenic developmental, thus modeling the disease and providing a potential tool to design therapeutic approaches for disorders of cartilage [112].

Nonetheless, the ultimate utility of iPSCs will be for drug screening and identification of novel pathways critical to rescue diseases. Dyskeratosis Congenita (DC) is a telomere disorder resulting in degeneration of multiple tissues types [125]. In their study, Agarwal *et al.* [126] reported that reprogramming DC somatic cells into iPSCs restores telomere elongation in these cells despite genetic lesions affecting telomerase, thus showing a rescue of the disease phenotype in DC cells. Furthermore, this study also indicated that activation of pluripotency-associated transcription factors like OSKM, which often target telomerase components (TERC) leading to the self-renewing characteristics of ESCs, may be a strategy therapeutically beneficial in DC patients. Activation of these transcription factors through reprogramming techniques in DC cells restored telomerase RNA component (TERC) levels and normal telomere maintenance. In this example, the use and derivation of iPSCs from patients exhibiting DC revealed a novel pathway resulting in the disease phenotype and provided a potential rescue, therefore suggesting, that drugs increasing TERC expression could be of therapeutic value to DC patients. Thus, iPSCs provide a method to bridge the gap between mouse models, which can be inaccurate or not translational, and human clinical studies, which are extremely expensive, time consuming and strictly regulated. In this context, the use of iPSCs to model and subsequently study disease pathology, small molecule screening and drug development, could represent a transforming tool in modern pharmacology.

## 5. Limitations and Concerns of Current iPSCs Technology

While recent research has made clear the potential application of iPSCs technology in translational medicine, there are still significant concerns associated with the reprogramming of adult somatic cells that must be addressed before the mainstream use of this technology. The long-term effects of an induced pluripotent state are still unclear. The changes associated with reprogramming, such as activation of oncogenes and tumor suppressor genes, the risk of cancer or teratocarcinoma formation and the lack of robust and effective differentiation protocols all pose significant challenges to the use of iPSCs as cell based therapies for human patients.

### 5.1. Functionality

One of the major obstacles hindering widespread use of iPSCs technology is the lack of efficient and at times complete differentiation of immature iPSCs to mature somatic cells needed for therapeutic purposes. Most current and successful studies have been centered on the differentiation and use of hematopoietic cells, neurons or cardiomyocytes—the easiest cells to differentiate from iPSCs. However, many other cell lines are of therapeutic value and current protocols do not always work, delivering low yield with significant differences in potency at maturity. Bao-Yang *et al.* [127] demonstrated that iPSCs displayed vast variability in their neural differentiation capacity, between 15%–79% as compared to 90%–97% from hESCs. It is important to note that through transcriptional analysis, the differentiation pathways were conserved between ESCs and iPSCs. Variability was maintained regardless of reprogramming technique, through multiple different iPSC lines, demonstrating the potential basis of these differences in potency to be the innate nature of the two types of pluripotent cell lines. This lack of robust and effective differentiation of iPSCs is highly concerning from a clinical standpoint, as the transplantation of immature cells could result in the development of cancers or teratocarcinoma. In a recent study signifying epigenetic memory could potentially improve differentiation procedures, Sanches-Freire *et al.* [128] demonstrated iPSCs derived from cardiac progenitors cells *vs.* fibroblasts showed increased efficiency in differentiation to cardiomyocytes, but remained functionally equivalent *in vivo*. Efforts toward improved differentiation protocols, potentially harnessing the utility of epigenetic programing as a form of “pre-conditioning”, could provide tangible solutions to the current limitations of iPSCs technology.

### 5.2. Chromosomal Aberrations and Genetic Modifications

From an outward appearance, iPSCs are thought to be analogous to ESCs, identical in make-up and functionality. Conversely, current understanding of both these pluripotent cell lines states otherwise. Advances in high-resolution genetic analysis has uncovered some subtle and not so subtle differences between these two cells lines. Current pluripotent stem cell technology, including both ESCs and iPSCs are greatly limited in their translational application due to fear of cancer development. Much is still unknown of the long-term effect of reprogramming methods, differentiation and expansion *in vitro*. Residual integration of reprograming components has limited iPSCs technology in cell-based therapy, though many efforts have been made towards generating non-intergrating reprograming protocols. Differentiation and expansion of these pluripotent cells lines is still dependent on *in vitro* culturing resulting in selective pressures for enhanced survival and proliferation. These concerns among many other chromosomal aberrations have severely hindered the use of iPSCs in clinical applications. Chin *et al.* [129], demonstrated that iPSCs have a novel and signature gene expression pattern unique from ESCs, but conserved between iPSCs derived from different species and reprograming techniques. Through a series of extensive analysis including array Comparative Genomic Hybridization (CGH), coding RNA profiling, miRNA profiling and histone modifications, this specific gene expression pattern found 3947 of 17,620 genes with differential expression levels between iPSCs and hESCs. Of these differences, 79% were suppressed; these genes correlated to energy production, RNA processing, DNA repair and mitosis. Furthermore, those upregulated in iPSCs relative to ESCs were implicated in differentiation [129]. These results are indicative of an inefficient silenced expression pattern of the parent somatic cell or insufficient induction of ESC genes, demonstrating genetic disparities between iPSCs and ESCs despite similarities *in vivo*. Further studies demonstrate that DNA methylation, a primary mechanism of epigenetic transcriptional regulation shows variation between iPSCs and hESCs [130]; iPSCs showed increased methylation as compared to hESCs implying differential gene regulation and epigenetics. The impact of these genetic changes is still unclear and many questions need to be answered before iPSCs are of use from a clinical perspective.

### 5.3. Cancer and Terotocarcinoma Formation

Cancer and/or the formations of teratocarcinomas are concern of iPSCs-based therapies. The introduction of cells of varying potency with manipulated gene expression is of great concern from the clinical perspective. For this reason, extensive studies must be conducted on the genetic and chromosomal aberrations that take place during reprogramming, *in vitro* expansion and differentiation. Through the technique of high resolution Single Nucleotide Polymorphism (SNP) Analysis, it was demonstrated that reprogramming of somatic cells to iPSCs is associated with the deletion of tumors suppressing genes during the time of differentiation, and expansion was associated with duplication of oncogenes [131]. Furthermore, full or partial chromosome aberrations, including a novel aneuploidy have been observed in early passage iPSCs. Mayshar *et al.* [132] reported a high incident of chromosome 12 duplications and significant enrichment of cell cycle-related genes implying limited differentiation and increase tumorigenicity. Lee *et al.* [133] demonstrated the disruption of control mechanism of Transposable Elements (TE) could facilitate retroactive transposons resulting in cancer. Similarly, Shukla showed a specific TE, Line-1 (L1) retro-transposons to be implicated in tumorigenesis in hepatocellular carcinoma. When Line-1 activity was compared in iPSCs to its parent cells, it was found to have increased by orders of magnitude, demonstrating reprogramming techniques significantly increase the risk of genetic instability and potential for cancer development [134].

### 5.4. Immunogenicity

Although it is widely assumed that autologous iPSCs and their derivatives should be immunologically tolerated by the recipient, the immunogenicity of iPSCs derivatives remains very controversial and quite complicated. The immunogenicity of iPSCs has been controversial since the original report that syngeneic mouse iPSCs elicited an immune response after transplantation [135]. Several studies suggest that both genetic and epigenetic defects occurring randomly during somatic reprogramming can directly or indirectly contribute to the immunogenicity of iPSCs derivatives [136,137,138]. Additional potential causes of iPSCs immunogenicity is immaturity of cells differentiated from iPSCs *in vitro* [139], and culturing of iPSCs, or their differentiated progeny, with xenogeneic or non-physiological culture reagents [140].

Zhao and colleagues found a subpopulation of cells derived from mouse iPSCs to be immunogenic, and the immune rejection T cell dependent [135]. In this study two genes were identified, *Hormad1* and *Zg16*, as abnormally expressed in iPSCs-teratoma but not in ES-teratoma, being directly responsible for the immunogenicity of iPSCs derivatives. In contrast, other studies suggested that the integration-free human iPSCs has low or negligible immunogenicity [141,142]. Morizane *et al.* [143] performed autologous iPSC-derived neural cell transplantation in a primate model and also found minimal evidence of chronic immune response in the brain. Although further confirmatory work is necessary, these latter studies are encouraging. In general, compared to allograft, the immunogenicity of autologous iPSC derivatives is much weaker. A recent study demonstrated that the immune response towards antigens of iPSCs is dependent on the immune environment of the transplantation site [144]. Understanding the risk of an immune response and developing strategies to minimize it will indeed be important steps before clinical application of iPSCs.

## 6. Concluding Remarks

The breakthrough discovery of iPSCs has greatly reshaped the scientific and political landscapes of stem cell biology. This discovery is a remarkable achievement for research and potential therapy. The ease of their derivation, capacity for rapid and robust expansion *in vitro*, ESC-like characteristics, minimal immune response due to patient matched cell lines and most importantly, their plentiful and uncontroversial source, make iPSCs an ideal replacement for hESCs in translational medicine. Because of fewer ethical restrictions and readily accessible donor tissue, additional research opportunities, both basic and translational, will be further performed to better understand the nature of iPSCs. However, as addressed in this review, recent research has brought to light significant differences between iPSCs and ESCs at the genomic level causing great concern in their translational application, particularly as cell-based therapies. During reprogramming, chromosomal aberrations can result in the deletion of tumor suppressor genes and duplication of oncogenes. These oncogenes are often highly selected for during *in vitro* expansion and differentiation [129]. Moreover, transplantation of these cells to human patients could lead to cancer, a significant limitation from a clinical perspective. It is not clear if reprograming techniques efficiently suppress parent somatic cell genes while inducing ESC genes. The result of this “incomplete” reprogramming is not fully understood, but thought to give varying levels of differentiation and potency to these cells. The transplantation of immature cells could again result in cancer or teratocarcinoma formation limiting their translational application. As improved reprogramming and differentiation protocols are developed, the use of iPSCs in disease modeling and drug discovery will be more applicable. Current techniques produce disease phenotypes, however, the extent of recapitulating all aspects of the disease is still limited. Overall, the vast potential of iPSCs in cell-based therapeutics, drug discovery and disease modeling is clear, though significantly hindered by the lack of understanding of the long-term risks. Until more is understood about the mechanisms of induced pluripotency and differentiation, and the development of extensive screening procedures for genetic aberrations resulting in stable genomes, the use of iPSCs technology remains inadequate for most translational medicine and clinical applications.
